# Improving classification of pollen grain images of the POLEN23E dataset through three different applications of deep learning convolutional neural networks

**DOI:** 10.1371/journal.pone.0201807

**Published:** 2018-09-14

**Authors:** Víctor Sevillano, José L. Aznarte

**Affiliations:** 1 Technical Superior School of Computer Engineering, Universidad Nacional de Educación a Distancia – UNED, Madrid, Spain; 2 Artificial Intelligence Department, Universidad Nacional de Educación a Distancia – UNED, Madrid, Spain; Fred Hutchinson Cancer Research Center, UNITED STATES

## Abstract

In palynology, the visual classification of pollen grains from different species is a hard task which is usually tackled by human operators using microscopes. Its complete automatization would save a high quantity of resources and provide valuable improvements especially for allergy-related information systems, but also for other application fields as paleoclimate reconstruction, quality control of honey based products, collection of evidences in criminal investigations or fabric dating and tracking. This paper presents three state-of-the-art deep learning classification methods applied to the recently published POLEN23E image dataset. The three methods make use of convolutional neural networks: the first one is strictly based on the idea of transfer learning, the second one is based on feature extraction and the third one represents a hybrid approach, combining transfer learning and feature extraction. The results from the three methods are indeed very good, reaching over 97% correct classification rates in images not previously seen by the models, where other authors reported around 70.

## Introduction

The surge in the prevalence of allergies, with 30 percent of adults and 40 percent of children having at least one allergy according to the ACAAI [1], implies that measuring the concentrations of airborne pollen grains becomes a necessity for public health institutions and patients. The determination of such concentrations is usually performed through volumetric spore traps which capture pollen grains, which in turn have to be visually identified and counted. The morphological similarity between pollen grains from different vegetal species complicates the identification process, which is usually performed by the visual inspection of optical microscope images by an experienced human operator.

During last decades, different computational intelligence or machine learning techniques have been developed to detect objects of interest in images and to identify categories of such objects (see [2] for a comprehensive review). Models built with different approaches and data sets, with different number of classes also. This work reflects the interest in finding a global classification method capable of adapting to different data sets.

These techniques try to teach computers to do what humans do naturally, i.e., to learn from experience, and use algorithms capable to learn directly from the images without previous knowledge of the field under study. A common approach is to extract discriminant features that represent particular characteristics of the objects of interest. These extracted features are then used to develop models capable to learn and identify patterns from the image data.

In the standard automatic classification approaches, the design and selection of these features is a time-consuming manual process which requires a deep mathematical knowledge of the information that can be obtained from images, as extraction of features involves pre-processing of the images by different operations to discriminate each of them. Recently, so-called *deep learning* algorithms have been developed to automatically learn these features from images without human intervention.

These algorithms are especially suited for image processing, and are being applied to solve problems such as facial recognition, motion detection, advanced driver assistance technologies such as autonomous driving, track detection, pedestrian detection and automatic parking, among many others. In [3], the authors use deep learning-based computer vision techniques to determine the make, model, and year of all motor vehicles encountered in particular neighborhoods. Data from this census of motor vehicles are used to accurately estimate income, race, education, and voting patterns at the zip code and precinct level. In other line of work, in [4] deep learning algorithms are used for detection of lymph node metastases from breast cancer. Also, in [5] deep learning techniques have been used to perform gene expression profiling as a tool to capture the gene expression patterns in cellular responses to diseases, genetic perturbations and drug treatments.

Given the similarities in the shape of different pollen types it is an interesting scientific challenge to study the use of deep learning techniques to develop automatic systems capable to distinguish between many different species of pollen.

In fact, over the last few years, many studies have been presented with the aim to ease the classification of pollen grains or to develop automatic classification methods. A few of the most recent make use of some forms of deep learning, and this is especially the case of the recent contribution presented in [6]. This contribution is threefold: the authors present a new, accessible and annotated pollen image dataset, they then study the performance of human operators faced to the pollen grain identification task and finally, they apply machine learning to solve this same problem. The results show no great improvement of the automatic techniques over the human operators.

However, in this paper, we present three automatic approaches based on deep learning that significantly increase the percentage of correct classifications on the same image dataset. The three methods rely on the use of a convolutional neural network (CNN) to automatically extract the discriminant features of the images. In the first method, a simple classifier is used to classify the images directly from the features extracted by the CNN. The second method applies a technique known as *transfer learning* and makes use of a pre-trained deep neural network. Finally, the third method constitutes a hybrid solution of the two previous.

The structure of the paper is as follows: the following section presents a brief description of the state of the art in automatic pollen classification. Consecutively, we present the image dataset and the deep learning methods, as well as the results obtained. These results are discussed, and some conclusions are drawn in closing.

## State of the art

As mentioned earlier, many authors have faced the task of pollen classification. Most of these authors propose approaches based on the analytical extraction of features from the images. Amongst them, we can distinguish at least three.

The first of these approaches is based on developing features using morphological methods in which visual features, such as shape, symmetry or size are measured. In this line of work, [7] works with grain perimeter, roundness and area, then using a Fisher linear discriminant to classify up to 12 different types of pollen collected on Henderson Island, Polynesia. They reported a proportion of correctly classified pollen that depends on the subset of variables used, with the best set of variables obtaining an overall classification rate of about 95%. [8] uses changes in grain contour to train a hidden Markov model as classifier to classify 17 genders and species from 11 different families of tropical honey bee’s plants reporting a mean of 98.77% of success. In [9], researchers use contour-inner pollen segmentation to classify a pollen collection dataset with 15 different types and 120 observations per type. A vector support machine was used as classifier, achieving a mean of 93.8% of accuracy.

A second approach is based on texture-based methods which make use of the characteristics of the grain surface as discriminant feature. For example, [10] used measurements of gray level co-occurrence matrices, neighborhood gray level dependence statistics and entropy to classify 5 different types of pollen from three locations, reporting a 76% of success. In [11], wavelet coefficients are used as a representation of the spatial frequency and to calculate a gray level co-occurrence matrix which is in turn used to classify 7 types of pollen reporting a F-score of 0.79. Researchers in [12] apply these same techniques to classify different pollen species responsible for respiratory allergies. The resulting system is evaluated for the discrimination of species of the Urticaceae family, which are quite similar. The performance reported is about 89% of correct pollen grains. Finally, in [13] an application of segmentation methods based on texture techniques are used to classify 5 different classes of Brazilian pollen, in which texture, shape and color features were extracted from each image, obtaining over 98% of success.

The third approach is constituted by hybrid methods which make a combined use of different features. For example, [14] combines morphological features, such as area and perimeter, with Fourier descriptors and color features to train a multilayer neural network as a classifier to identify 17 pollen species, reporting a mean of 96.49% of success. [15] also classifies five pollen types by using shape (area, perimeter, diameter) and texture features (mean, standard deviation, and the entropy of gray level histograms). The approach classifies fraudulent microscopic pollen grain objects with a reported 92.3% of success. In [16], authors propose an approach for performing automatic species-level recognition of fossil pollen grains in microscopy images that exploits both global shape and local texture characteristics in a patch-based matching methodology. The method introduces a criteria for selecting meaningful and discriminative exemplar patches. The technique uses these exemplars as a dictionary basis, and proposes a spatially-aware sparse coding method to match testing images for identification while maintaining global shape correspondence, achieving 86.13% accuracy on a difficult fine-grained species classification task, distinguishing three types of fossil spruce pollen. The same researchers, in [17], use a nearest‐neighbor instance‐based supervised layered learning system based on kernel density estimation capable of discriminating between the morphologically similar pollen of black and white spruce, achieving over 93% of accuracy in classification of congeneric species.

However, there is another way of tackling the problem of pollen image classification: instead of analytically extracting features from the images, one can rely on an automated system to do that job. An example of such alternative is presented, for example, in [18], where a model learns not only the features but also the classifier itself from training data under a deep learning framework. To further enhance the classification ability, the proposal makes use of transfer learning to leverage knowledge from networks that have been pre-trained on large datasets of images, to train a dataset of 30 pollen types, achieving a 94% correct classification rate. In [6], which presents and makes use of the same dataset used in this paper, three different alternatives based on automatic feature extractors are explored. The three feature extractors are the “bag of visual words” (BOW), the “color, shape and texture” (CST) and a combination of BOW and CST (CST+BOW). They test several machine learning classifiers, including two variations of support vector machines, a decision tree-based classifier and the *k*-nearest neighbor approach. According to these authors, the highest correct classification rate, 64%, is achieved using CST+BOW and support vector machines. Another example is [19], which uses a neural network approach to perform pollen classification for the reconstruction of remote paleo environments, reporting over 90% of the grains being correctly identified. BOW-related techniques have also been applied in [20], with a 70% reported correct classification rate over 9 pollen types, and in [21], with a reported 97.2% of accuracy over just 1 pollen variety.

When comparing different automatic classification methods, it is important to bear in mind that different experiments have different degrees of difficulty. Of course, the similarity of the grains from different species is the main challenge, but the quality of the images, for example, is crucial as well to develop good classifiers. Also, the amount of tagged images is essential, as more images for each type will allow to train much more accurate models. Finally, it is very different to classify just one pollen type with a binary classifier than developing a multiclass classifier able to recognize tens of pollen types. Thus, in the above comparison of the results reported by different authors we need to take into account these issues. For example, many of the aforementioned studies have been made with sets of data with a very limited amount of images or pollen types. For example, [10–13, 15, 20, 21] work with sets of data containing less than 10 pollen types. The results presented in [6–9, 14, 18] work with larger sets of data. Particularly [18] presents results over 94% for a set of images containing 30 different pollen types. Of course, as the sets contain more classes, the classification process becomes more complicated, presenting lower performances as in [6]. Attempts to classify datasets with large classes are a major challenge since they are intended to provide a more generalized method, applicable to a wider range of solutions.

The solution presented in this paper aims to provide a model capable of adapting to different data sets that contain a significant number of classes, even with similar features.

## Materials and methods

### Image pollen dataset

In [6] the authors presented an image dataset, called POLEN23E, which consists of photos of 23 pollen types present in the Brazilian savannah: *Anadenanthera colubrina*, *Arecaceae*, *Arrabidaea*, *Cecropia pachystachya*, *Chromolaena laevigata*, *Combretum discolor*, *Croton urucurana*, *Dipteryx alata*, *Eucalyptus*, *Faramea*, *Hyptis*, *Mabea fistulifera*, *Matayba guianensis*, *Mimosa somnians*, *Myrcia*, *Protium heptaphyllum*, *Qualea multiflora*, *Schinus terebinthifolius*, *Senegalia plumosa*, *Serjania laruotteana*, *Syagrus*, *Tridax procumbens* and *Urochloa decumbens*.

**Fig 1:**
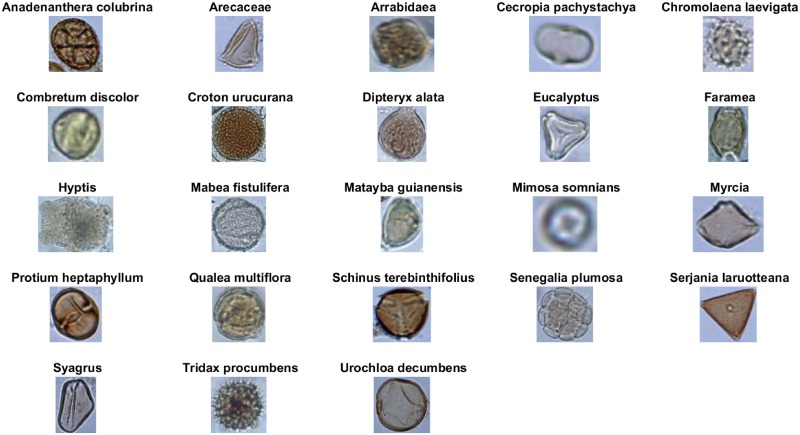
Sample images for each pollen type.

The POLLEN23E dataset is publicly available and, according to its description in [6], contains 35 sample images for each pollen type. These were taken with a digital microscope at different angles, compiling a total of 805 images. However, for the type *Anadenanthera colubrina* only 20 images were found in the original source, so in our experiment 15 synthetic images were generated through rotating and scaling the original images of this type. Fig 1 contains a sample of each species.

To assess the robustness of the models against over-fitting, the accuracy on each conducted experiment was measured using a 10 fold cross-validation process.

### Convolutional neural networks for image classification

The work presented in this paper is based on the automatic extraction of discriminant features from images by deep learning convolutional neural networks (CNN).

This type of network is a variation of the well known multilayer perceptron, however, because its application is performed in two-dimensional matrices, they are effective for artificial vision tasks, like image classification and segmentation, among other. The fundamentals of CNN are based on the Neocognitron, introduced by Kunihiko Fukushima in 1980 [22]. This model was later improved by Yann LeCun in 1998 [23], introducing a learning method capable to train the network through backpropagation. In the year 2012, they were refined by [24] and implemented in a GPU, thus obtaining impressive results.

The term “deep” usually refers to the number of hidden layers in the neural network. Traditional neural networks (as the multilayer perceptron) contain only two or three hidden layers, while deep networks can have hundreds of them. In order to train these networks, extensive tagged data sets are required. CNN are amongst the most popular types of deep neural networks.

**Fig 2:**
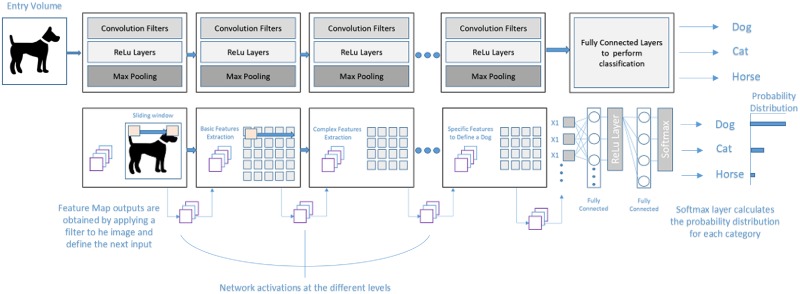
Convolutional neural network architecture and operation. Image based on a similar figure published in [26].

A CNN is especially suited for processing 2D data such as images as it makes use of 2D hidden (“convolutional”) layers to convolve the features with the input data. The main strength of CNN is that it eliminates the need for manual feature extraction by automatically extracting the more discriminant features of a set of images. Of course, this automated feature extraction makes CNN very useful for artificial vision tasks such as object classification. In this paper, only a succinct description of CNN is included. For more information, refer for example to [25]. Fig 2 shows a scheme of a CNN with many layers. Convolutional filters are applied to each training image with different resolutions, and the output of each convoluted image is used as input for the next layer. As shown in the figure, a CNN with a single convolutional layer consists of four fundamental components: the convolutional layer, a ReLu layer, a pooling layer and a fully connected layer.

The convolutional layer forms the basis of the neural network and performs the convolution operation on the input image. It consists of a three-dimensional array of neurons as a stack of two-dimensional layers of neurons, one for each channel.

The convolution operator has the effect of filtering the input image with a previously trained kernel. This transforms the data in such a way that certain features (determined by the kernel shape) become more dominant in the output image as these have a higher numerical value assigned to the pixels representing them. These kernels have specific image processing skills. An example is edge detection, that can be performed with kernels that highlight the gradient in a particular direction.

Neural networks are known to have some tolerance to small perturbations in the input data. For example, if two almost identical images (different only by a few pixels) are fed through a neural network, the result should be essentially the same. In the case of CNN, this is obtained, in part, by the reduction of sampling. By reducing the resolution, the same characteristics will correspond to a larger activation field in the input image. This is achieved through max-poolingoperations, which are very effective in summarizing characteristics about a region.

#### Training and using convolutional neural networks

To train a CNN from scratch, a very broad tagged data set must be collected, and a network architecture is designed to learn the discriminant features and distinguish the images. This is useful for new applications or applications that will have a very large number of output categories. This approach is less common because, due to the large amount of required images and the speed of learning, with present day hardware it usually takes a very long time to train these networks.

However, it is possible to apply a transfer learning (TL) approach, a process that involves the fine-tuning of a previously trained CNN. In this case, the process starts with a pretrained network (such as AlexNet [27] or GoogLeNet [28]), and feed them with new data containing previously unknown classes. After making some adjustments in the network, it is possible to perform a new task categorizing the new data provided to the network. This also has the advantage of needing much less data (thousands of images are processed instead of millions), so the calculation time is reduced to hours or minutes.

A less common but more specialized approach is the use of a CNN as feature extractor. Because all layers have assigned tasks related with learning certain features of the images, these features can be extracted from the network at any time during the training process. In standard operation, the CNN will use these features in its last layers, which work as an standard neural network classifier. However, these features can then be used as input for another machine learning classifier which will learn them to classify new data.

In this work we will implement three models of deep learning based on these CNN techniques. One of them will be strictly based on the idea of transfer learning, a second model will be based on feature extraction and a third hybrid model will combine TL followed by feature extraction.

### Linear discriminant classifier

When using a CNN as a feature extractor, as described above, it is important to devise a machine learning classifier which will take upon the extracted features and discriminate the input images according to them. In this paper we will use a linear discriminant (LD) classifier for this task.

Linear Discriminant Analysis is normally used as a technique for dimensionality reduction in machine learning applications. The objective is to project a dataset onto a lower-dimensional space with better class-separability. This technique was first formulated by Ronald A. Fisher in 1936 [29], and it also has some practical uses in classification problems. The LD approach is similar to a principal component analysis, but in addition to finding the component axes that maximize the variance of the data, we are also interested in finding the axes that maximize the separation between multiple classes.

In brief, the goal of LD is to project a feature space (a set of *n*-dimensional samples) onto a smaller subspace *k* (where *k* ≤ *n* − 1) while keeping the class-discriminatory information. In other words, a LD classifier explicitly attempts to model the difference between the classes of data. In general, dimensionality reduction does not only help reducing computational costs for a given classification task, but it can also be helpful to avoid overfitting by minimizing the error in parameter estimation.

LD analysis is closely related to variance analysis (ANOVA) and regression analysis, and is a popular classification algorithm because it is fast, accurate and easy to interpret. It is also especially suitable for large data sets, and it assumes that different classes generate data based on different Gaussian distributions. To train the classifier, the fitting function estimates the parameters of a Gaussian distribution for each class, then creating linear boundaries between classes. It is fast predicting, uses small memory and is easily interpretable, although it has comparatively low flexibility.

### Experimental design

To avoid overfitting and to test the robustness and generalization ability of the proposed methods, a 10 fold cross-validation scheme has been applied. For each fold, the original dataset has been divided into two subsets of images: a training set and a test set, containing 90% and 10% of the observations respectively, reserving the last one to test the models after the training process. Since there are 35 images per pollen type, in each fold the training set contains 32 images and the test set is composed of 3 images.

From the previously described techniques, we implemented and trained the following combinations.

#### Setup A: Feature extraction and linear discriminant classifier (FE+LD)

This approach is based on the idea of taking advantage of a pre-trained convolutional neural network architecture (in our case, AlexNet) to perform feature extraction, and then use a LD classifier upon these features.

The AlexNet network builds a hierarchical representation of the images. Deeper layers hold sets of higher level extracted features, constructed from lower level extracted features from previous layers. Thus, the first layer of the network consists of convolutional filters which learn the basic features. These primitive features are then processed by deeper layers of the network, combining the first features to create new ones on a higher level. This higher level features are more suitable for classification tasks since they combine all the primitive features in a better representation of the image. Selecting a particular layer to extract features is a design consideration, but it is common to select the layer immediately before the classification layer. We have adopted this convention in Setup A, selecting the seventh convolutional layer of AlexNet to obtain the representative features.

**Fig 3:**
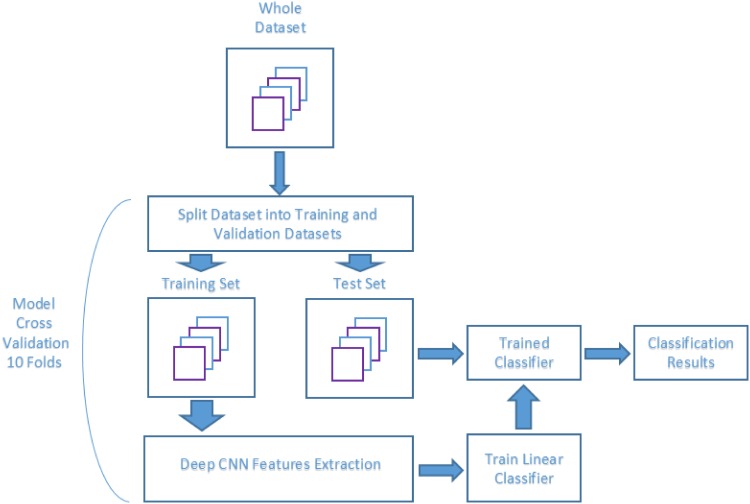
Cross-validation schematic for setups A and C.

Fig 3 shows how the aforementioned cross-validation scheme has been applied in this setup. For each of the 10 folds, the dataset is initially divided into two subsets, training and test. Each training set is used to perform the feature extraction process. The resulting set of extracted features is used to train the LD classifier. Once the classifier has been trained with these extracted features, it is used to classify the images of the test set reserved at the beginning of the procedure.

#### Setup B: Transfer learning (TL)

In this setup, the pre-trained CNN AlexNet has been adjusted to learn the particular features of the POLLEN23E images dataset. The last three layers of the AlexNet are originally configured for 1000 classes, and they must be fine-tuned for the new classification problem. For this purpose, all layers are extracted, except the last three, from the pre-trained network and transferred to learn the new classification task by replacing these last three layers with a new fully connected layer, a softmax layer, and a classification output layer, all adjusted to 23 classes. Then the network is retrained to learn the new task.

The idea is to keep the features pre-learnt in the convolutional layers of the AlexNet and force the learning just in the fully connected ones. Hence, we set a low learning rate (0.0001) in the transferred layers to fine-tune them and a higher learning rate (0.2) in the fully connected layers to allow learning in the newly created layers. This combination of learning rate settings results in fast learning only in the new layers and slower learning in the early ones.

**Fig 4:**
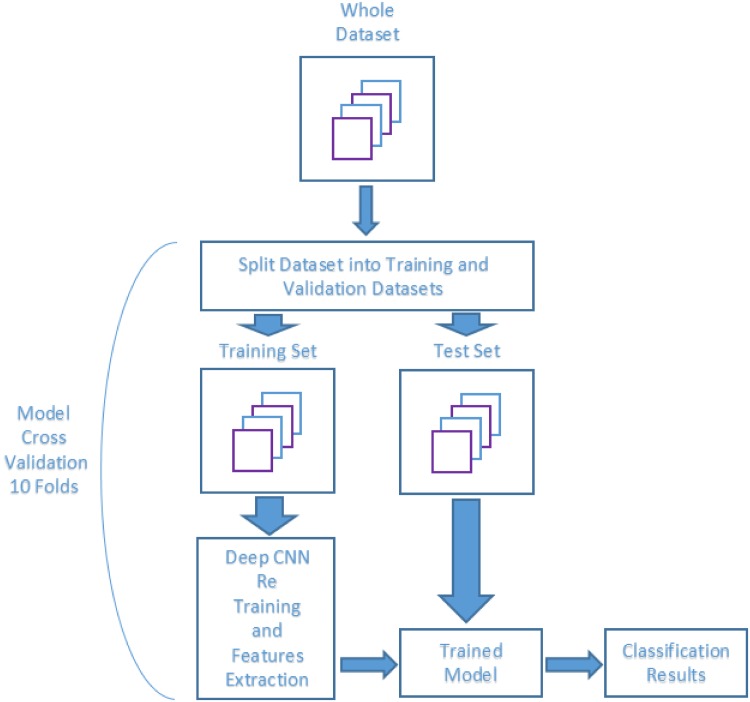
Cross-validation schematic for setup B.

Fig 4 shows the schematic description of the cross-validation process applied in this setup to retrain the CNN and extract the features from the training dataset, and then apply the retrained network to the test set.

#### Setup C: Transfer learning, feature extraction and linear discriminant (TL+FE+LD)

This setup is a hybrid approach aimed to preserve the advantages of the two previous setups. As stated above, through transfer learning we retrain the last three layers of the network leaving the previous layers practically unchanged by setting a very low learning rate for them. However, during the training process, it is observed that even a low learning ratio for these previous layers of the network is sufficient to modify the weights of the different convolutional filters, allowing them to incorporate features of the new provided images, and thus increasing the classification performance. Then, once applied transfer learning, it is possible to extract the features from the fine-tuned CNN and subsequently apply a classifier.

In this approach, the learnt features can be extracted from the layer immediately before the classification layer, exactly as we did in setup A. However, we can also extract the features from the fully connected layer or even the softmax layer recently retrained. For a dataset like POLEN23E, with a reduced amount of observations, it is common to select the first option, that is, extracting the features from the layer immediately before the classification layer. In this case, the convolutional filters from the earlier layers will have slightly changed their weights adding new features, but keeping most of the original network features. We keep the same learning rates applied in setup B, using a low learning rate (0.0001) in the transferred layers and a higher learning rate (0.2) in the fully connected layer. For sets of images with a higher amount of observations, extracting features from the new convolutional layers can be a good alternative to increase the performance.

Once the network has been re-trained and the features extracted, a linear discriminant classifier is built to tell the images apart.

As it was the case with setup A and B, a 10-fold cross-validation procedure has been applied. The schematic of this procedure for setup C is the same as for setup A, shown in Fig 3.

## Results

This section presents the results obtained in the experiments. As mentioned above, these experiments correspond to the application of three different setups based on different combinations of techniques. To compare the results of the three setups amongst them and with previous works, we use the following commonly used performance measures for classification:

$$\begin{array}{c} \text{CCR}=\frac{TP+TN}{TP+TN+FP+FN} \end{array}$$ (1)

$$\begin{array}{c} \text{precision}=\frac{TP}{TP+FP} \end{array}$$ (2)

$$\begin{array}{c} \text{recall}=\frac{TP}{TP+FN} \end{array}$$ (3)

$$\begin{array}{c} \text{F-score}=2\cdot \frac{\text{precision}\cdot \text{recall}}{\text{precision}+\text{recall}} \end{array}$$ (4)

In all these, *TP* refers to true positives, *TN* to true negatives, *FP* to false positives, and *FN* to false negatives.

In machine learning, it is important to compare models using data previously unseen during the training process. These data constitute an example close to reality, since they are completely unknown to the models and allow to measure their behavior against new observations. Table 1 presents the results obtained by the three setups using the test datasets. This table shows averages of the cross-validation results for the correct classification rate (CCR), precision, recall, and F-score.

**Table 1:** Average correct classification rate over the test set for each setup. Results from [6] are computed from their confusion matrices. In parentheses under the values, standard deviation.

	CCR (%)	precision	recall	F-score
CST+BOW (from [6])	68.5714	0.3988	0.8203	0.5366
Human (from [6])	63.5710	0.3030	0.6279	0.4087
Setup A (FE+LD)	96.6247 (± 1.1107)	0.9366 (± 0.0210)	0.9955 (± 0.0016)	0.9592 (± 0.0137)
Setup B (TL)	96.1529 (± 1.2089)	0.9275 (± 0.0229)	0.9949 (± 0.0020)	0.9541 (± 0.0151)
Setup C (TL+FE+LD)	97.2273 (± 0.9000)	0.9477 (± 0.0170)	0.9964 (± 0.0014)	0.9669 (± 0.0115)

**Fig 5:**
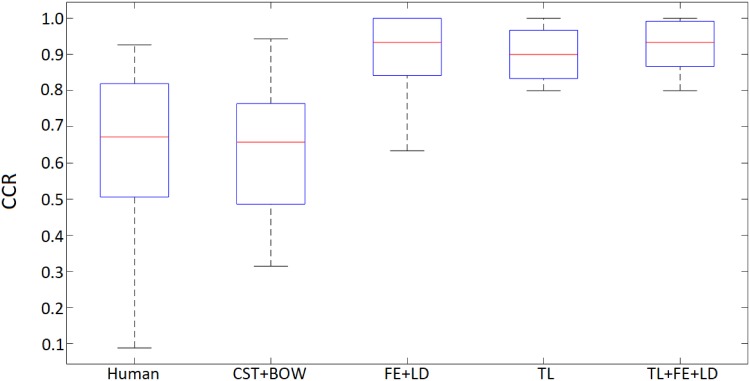
Correct classification rate for human operators and the best model reported by [6], together with the three proposed deep learning setups.

From the table it is clear that Setup C obtains the best results in all indices. A very high CCR value, 97.2273%, is obtained with a low standard deviation of 0.9000%. This is accompanied by high precision and recall values which verify its good performance against false positives and false negatives, also showing a high F-score value (0.9669) with a low standard deviation (0.0115). The comparison with the results from [6] show that our models improve especialy in precision, that is, in the proportion of true positives with respect to the total of predicted positives. Recall is also improved, especially from the human-based classification results. The results for CCR are displayed in Fig 5.

In this figure, we observe that setup C is sligthly better both in terms of median CCR and in the dispersion of this magnitude through the different cross-validation folds. The bottom “whisker” for this setup is located above 80%, meaning this is the minimum CCR we can expect for any class. The median is clearly above the value of setup B and slightly better than the value obtained by setup A. However, the latter bottom “whisker” places the minimum near 0.6 and very far from the first quartile, which suggests discarding this model as a solution. Setup C is also superior to setup B in terms of the first and third quartiles. However, a Wilcoxon signed-rank test shows that there are no significant differences amongst the results of the three methods, so any of them could be used.

**Fig 6:**
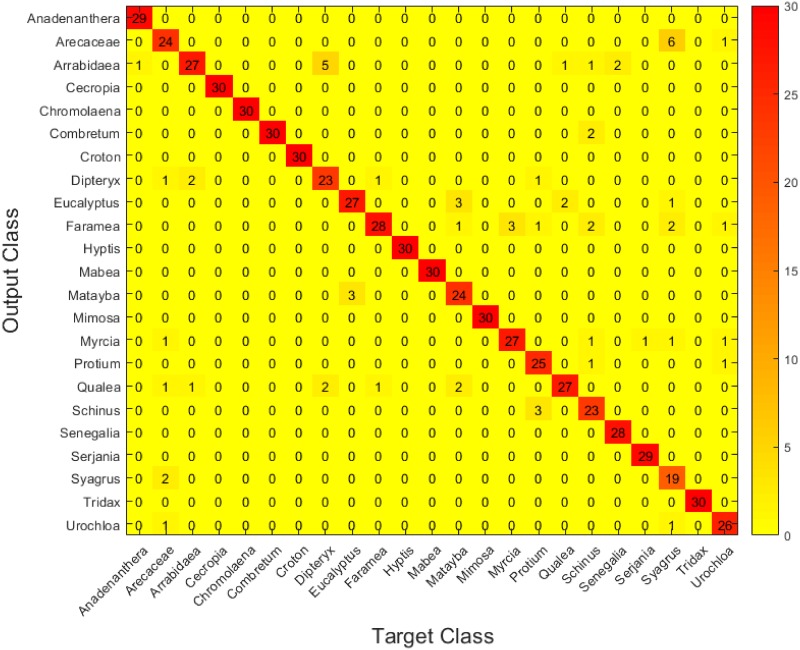
Confusion matrix for the test set in setup A.

**Fig 7:**
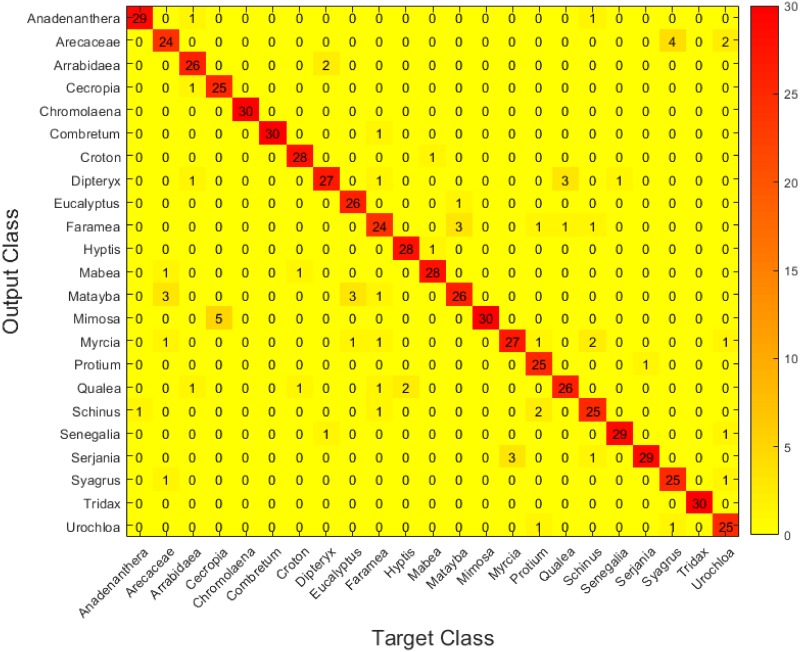
Confusion matrix for the test set in setup B.

**Fig 8:**
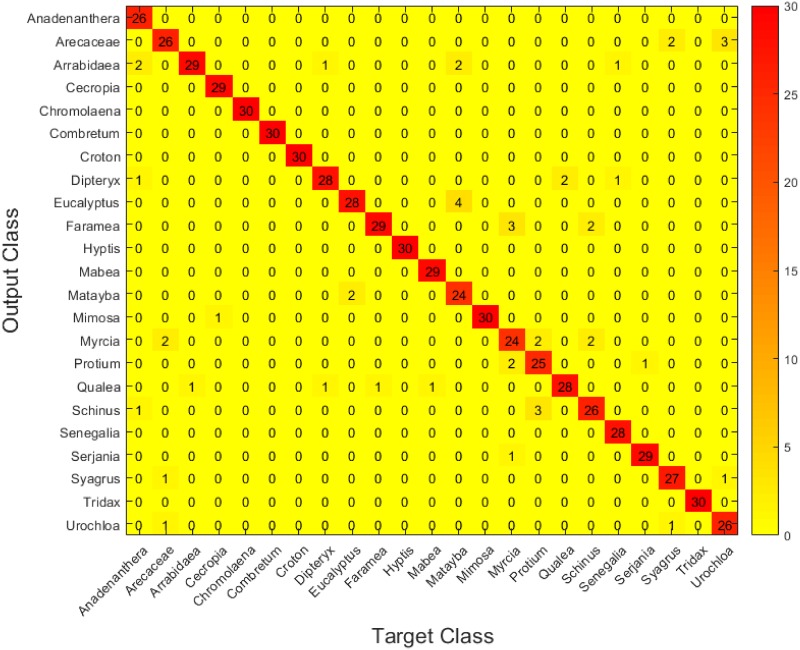
Confusion matrix for the test set in setup C.

Figs 6, 7 and 8 show the confusion matrices for the three setups. These matrices have been constructed by accumulating the individual cross-validation results in the test dataset(3 test images per fold, hence the total of 30 in the diagonals) Again, it can be easily observed that setup C shows slightly better performance than the other two.

Concretely, we can see how the three setups manage to identify most of the pollen types correctly, and it is interesting how the three find more difficulties when trying to discriminate certain types that are, undoubtly, similar: *Arecaceae*, for example, is usually confused with *Syagrus* and *Urochloa decumbens*, while *Matayba guianensis* is confused with *Eucalyptus*. However, it is interesting how setup B has trouble distinguishing *Mimosa somnians* and *Cecropia pachystachya*, while the other two are capable of discriminating them correctly. Also, setup A gets not so good results when trying to separate *Arrabidaea* from *Dipteryx alata*, while the other two (especially setup C) have no problems with these two types. These results show that each setup has its own strengths and weaknesses in this framework, and are in accordance with the Wilcoxon test mentioned above.

To compare the computational complexity of each setup, Table 2 presents the prediction speed (measured in observations processed per second) and the total training time for each setup. We observe that the three models offer reasonable training times, highlighting the setup C prediction speed, with approximately 170 predictions per second.

**Table 2:** Measures for computational complexity.

	Prediction speed	Training time
Setup A (FE+LD)	∼140 obs/sec	8.69 min
Setup B (TL)	∼155 obs/sec	16.49 min
Setup C (TL+FE+LD)	∼170 obs/sec	16.61 min

## Discussion

In this paper, we present three deep learning classifiers of pollen grain images, based on a pre-trained convolutional neuronal network (Alexnet) to which we apply transfer learning and extract features that are subsequently classified by a linear discriminant classifier.

Any of the three hereby proposed solutions considerably improve the results presented in [6] for the same POLEN23E dataset. In that work, researchers obtain the best results by applying a technique known as bag-of-words (BOW) as feature extractor. However, the results obtained by this technique are poor, with a median CCR around 66% for the best non-human classifier. Humans reached a 67% median CCR in their experiment.

To explain these results, the nature of the POLLEN23E images must be taken into account. These images are centered on the frame and occupy most of it. In addition, there are similarities between different classes that make the classification process difficult by itself. The BOW technique is used to classify images, but is generally better suited for images that contain different features and are not centered inside the frame (landscapes or objects of a very diverse nature). This has to do with the fact that BOW is based on a *k*-nearest neighbor algorithm, which makes it outstand, for example, in handwritten digit recognition because the space of digit images (and thus the possible variation amongst them) is much smaller than the space of all possible images.

However, for the POLEN23E dataset, features are spread almost uniformly throughout the feature space, not concentrated on or near any lower dimensional manifold. Hence, given the high similarity of the images, many of the features that the *k*-nearest neighbor algorithm must classify will be very similar. On the other hand, BOW extracts by default 500 “words”, generating a high dimensionality problem for the classifier. Considering that many of these “words” are similar because of the images similarity, even amongst different classes, it incurs in what is known as the curse of dimensionality. As mentioned in [30], with this setup, in high dimensions all examples look alike, implying that the choice of the nearest neighbor (and therefore the class) tends to be random.

The solution proposed in this paper is based on deep learning convolutional filters. These filters are capable of extracting specific and discriminating features of each image to work properly with a simple classifier in high dimensions. The way in which these filters extract the image features makes possible to obtain the most relevant characteristics to each class, avoiding those common to several classes that introduce noise in the classifier. Therefore, the instances (features) are sufficiently distant between their corresponding classes for the classifier to correctly identify them.

**Fig 9:**
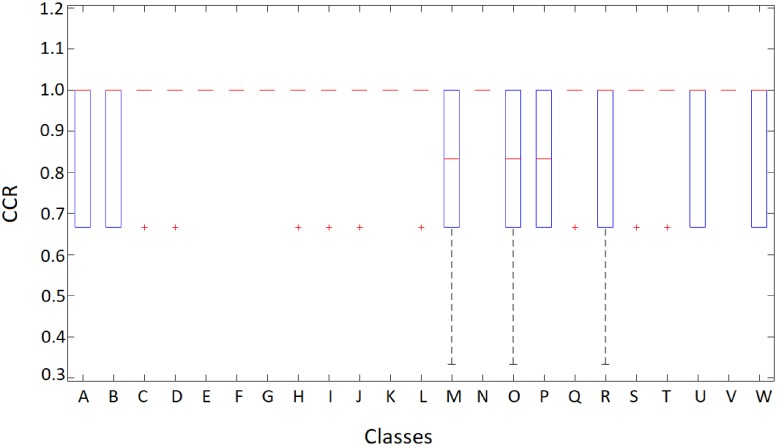
Distribution of the correct classification rate for the test set in setup C, by pollen type. Types: A) *Anadenanthera colubrina*, B) *Arecaceae*, C) *Arrabidaea*, D) *Cecropia pachystachya*, E) *Chromolaena laevigata*, F) *Combretum discolor*, G) *Croton urucurana*, H) *Dipteryx alata*, I) *Eucalyptus*, J) *Faramea*, K) *Hyptis*, L) *Mabea fistulifera*, M) *Matayba guianensis*, N) *Mimosa somnians*, O) *Myrcia*, P) *Protium heptaphyllum*, Q) *Qualea multiflora*, R) *Schinus terebinthifolius*, S) *Senegalia plumosa*, T) *Serjania laruotteana*, U) *Syagrus*, V) *Tridax procumbens*, W) *Urochloa decumbens*.

Fig 9 shows the cross-validation average CCR obtained by setup C *for each class*. It is important to clarify that, since in each fold the test set was composed only of 3 images, the CCR for each class in each fold could take values in $\left\{1,0.\bar{66},0.\bar{33},0\right\}$, depending on the model properly identifying all the 3 images, two of them, one or none at all (the latter has not been recorded in our experiments).

We note that there are three classes (*Matayba guianensis*, *Myrcia* and *Schinus terebinthifolius*) which registered a low CCR value of $33.\bar{3}\%$ in some of the folds, while the same classes reach CCR values of 100% in other folds. This surely happens because the number of observations contained in the POLEN23E set is small, making the results of each fold very sensitive to the training/test split of the dataset. If the images included in the test set are “difficult”, for example in the sense that the grains are in such position that some of their distinguishing characteristics are not clearly seen, the model performance will be easily affected. This effect could be diminished by increasing the number of available observations per class, allowing for bigger test sets which would make the experiments more robust.

It is also remarkable that the proposed setup manages to identify 100% of the examples in almost all of the folds for two thirds of the available classes. For these fifteen classes (*Arrabidaea*, *Cecropia pachystachya*, *Chromolaena laevigata*, *Combretum discolor*, *Croton urucurana*, *Dipteryx alata*, *Eucalyptus*, *Faramea*, *Hyptis*, *Mabea fistulifera*, *Mimosa somnians*, *Qualea multiflora*, *Senegalia plumosa*, *Serjania laruotteana* and *Tridax procumbens*) the first and third quartiles are equal to the median, which is set at 100%. They are thus perfectly identified by the model, which is able to fully aprehend the particular characteristics of each class.

In a similar take, in [18] an approach based on pre-trained CNN transfer learning is presented, obtaining results around 94% of CCR for data sets obtained by light-microscopy and scanning electron microscopy. However, they do not specify if the results are obtained over the training set or the test set, and hence they are not directly comparable with ours. They also use data augmentation to increase the number of samples for their datasets, a technique that could probably improve our results as well.

## Conclusions and future work

In this work, three different models based on deep learning convolutional neural networks and capable of satisfactorily classifying the POLEN23E dataset images have been presented. The three solutions have exceeded the 95% threshold of correct classifications. Amongst them, the most accurate is obtained after applying transfer learning to the AlexNet pre-trained network and then using a linear discriminant classifier to the extracted features. This solution perfectly classifies two thirds of the pollen types, reaching up to a global 97% of correctly classified samples. Finding an automated solution to pollen classification implies significant potential advantages in different areas, including allergy-related clinical applications or industries like honey production.

These excellent results prompt us to try the same ideas with different datasets and different pre-trained networks, and also networks trained from scratch. Expanding the number of images available through data-augmentation also looks promising in order to increase the classification capacity of the proposed solutions.
